# Wnt Secretion and Gradient Formation

**DOI:** 10.3390/ijms14035130

**Published:** 2013-03-01

**Authors:** Gonzalo P. Solis, Anne-Marie Lüchtenborg, Vladimir L. Katanaev

**Affiliations:** 1 Department of Pharmacology and Toxicology, University of Lausanne, Rue du Bugnon 27, Lausanne CH-1005, Switzerland; E-Mail: Anne-Marie.Luchtenborg@unil.ch; 2 Institute of Protein Research, Russian Academy of Sciences, Institutskaya 4, Pushchino 142290, Russia

**Keywords:** Wnt secretion, morphogen, gradient formation, reggie/flotillin proteins, breast cancer, *Drosophila*

## Abstract

Concentration gradients formed by the lipid-modified morphogens of the Wnt family are known for their pivotal roles during embryogenesis and adult tissue homeostasis. Wnt morphogens are also implicated in a variety of human diseases, especially cancer. Therefore, the signaling cascades triggered by Wnts have received considerable attention during recent decades. However, how Wnts are secreted and how concentration gradients are formed remains poorly understood. The use of model organisms such as *Drosophila melanogaster* has provided important advances in this area. For instance, we have previously shown that the lipid raft-associated reggie/flotillin proteins influence Wnt secretion and spreading in *Drosophila*. Our work supports the notion that producing cells secrete Wnt molecules in at least two pools: a poorly diffusible one and a reggie/flotillin-dependent highly diffusible pool which allows morphogen spreading over long distances away from its source of production. Here we revise the current views of Wnt secretion and spreading, and propose two models for the role of the reggie/flotillin proteins in these processes: (i) reggies/flotillins regulate the basolateral endocytosis of the poorly diffusible, membrane-bound Wnt pool, which is then sorted and secreted to apical compartments for long-range diffusion, and (ii) lipid rafts organized by reggies/flotillins serve as “dating points” where extracellular Wnt transiently interacts with lipoprotein receptors to allow its capture and further spreading via lipoprotein particles. We further discuss these processes in the context of human breast cancer. A better understanding of these phenomena may be relevant for identification of novel drug targets and therapeutic strategies.

## 1. Introduction

Signaling cascades triggered by the lipid-modified morphogens of the Wnt family are involved in virtually all aspects of development throughout the animal kingdom. Specifically, Wnt signaling is essential for regulation of cell proliferation and differentiation, cell polarity and migration, and development of the cardiovascular, nervous and mammary systems [1]. The Wnt signaling is mostly silent during adult life. Therefore, it is not surprising that its improper activation causes a wide range of human diseases, most notably cancer [2–4]. Although the Wnt-mediated signaling pathways have been extensively studied during the last decades, much remains to be learned about how Wnts are secreted and how extracellular morphogen gradients are formed. A better understanding of these essential cellular processes may have a great impact for development of therapies needed to combat cancer and other diseases in which Wnt signaling is involved. Here, we outline recent advances in understanding of the Wnt secretion and spreading in polarized epithelia, and we particularly focus on the role of the lipid raft-associated reggie/flotillin proteins in these processes. Additionally, we briefly discuss their potential implications in breast cancer.

## 2. Wnt Proteins and Signaling

Wnts represent a large family of secreted proteins highly conserved among vertebrates and invertebrates [5]. In the human genome, 19 Wnt genes have been identified: Wnt1, 2, 2b, 3, 3a, 4, 5a, 5b, 6, 7a, 7b, 8a, 8b, 9a, 9b, 10a, 10b, 11 and 16 [5]. Additional to this complexity, two branches of signaling by Wnt ligands have been described: the β-catenin-dependent “canonical” pathway and the β-catenin-independent “non-canonical” pathway [6]. The canonical pathway is triggered by the Wnt binding to and activation of a receptor complex including the transmembrane proteins Frizzled and LRP5/6. This receptor complex transduces the signal to several intracellular proteins resulting in inhibition of the so-called “β-catenin destruction complex” [1,7]. Cytoplasmic β-catenin levels are regulated through its continuous proteasome-mediated degradation induced by this complex. When cells receive a Wnt signal, the degradation is inhibited and β-catenin accumulates in the cytoplasm and nucleus. Nuclear β-catenin interacts with transcription factors such as LEF/TCF and regulates transcription of various Wnt target genes essential for cell proliferation, cell fate specification and differentiation [1]. On the other hand, β-catenin-mediated transcription is not involved in the non-canonical Wnt signaling. Although less well studied, it is clear that the non-canonical signaling regulates cell migration and organ morphogenesis through the activation of Ca^2+^ signaling and cytoskeletal proteins including the small GTPases RhoA, Rac1 and Cdc42, and the kinases ROCK, PKC, and JNK [6]. Some secreted Wnt proteins such as Wnt1, 3, 3a, 8a and 8b are described to activate exclusively the canonical signaling, whereas Wnt5a, 7a, 7b and 11 have mostly been implicated in the non-canonical pathway [6]. However, the ability of a given Wnt to activate the canonical *vs* the non-canonical pathway may to a large extent depend on the Frizzled receptor repertoire expressed by a given cell type [8].

## 3. Wnt Secretion and Gradient Formation

Wnt ligands are produced and secreted by a defined subset of cells (“Wnt-producing cells”) and then spread through the tissue to form a concentration gradient. “Wnt-receiving cells” in the vicinity of the Wnt-producing cells respond in a concentration-dependent manner by modulating the expression of the target genes [1]. Genes activated close to the source of Wnt production are called “short-range target” genes, whereas genes activated at longer distances from the Wnt-producing cells are recognized as “long-range target” genes. Therefore, formation of extracellular concentration gradients is a crucial feature in Wnt response programs. How Wnt ligands are secreted and how Wnt gradients are formed is currently not well understood and very divergent models explaining these features have been proposed [6,9,10]. In addition to initiation of the Wnt-response programs, the receiving cells play a role in Wnt gradient formation by endocytosis and lysosomal degradation of the morphogen [11,12].

The collected experimental evidence for Wnt secretion indicates existence of specialized machineries for intracellular transport of Wnt proteins, discarding the idea of a passive flow of Wnts through the secretory pathway. Almost all secreted Wnts analyzed so far display high hydrophobicity due to the acylation of conserved amino acid residues by the acyltransferase Porcupine in the endoplasmatic reticulum [9]. Besides acylations, secreted Wnts are also modified by *N*-glycosylations. Both types of post-translational modifications are relevant during Wnt signaling, but they are also important for proper Wnt protein folding and intracellular trafficking [9]. At the Golgi, Wnts are escorted to the plasma membrane (PM) by an evolutionary conserved multipass transmembrane protein called Wntless/Evenness interrupted/Sprinter [13–15] (hereafter-Wntless) which specifically interacts with acylated Wnts [16]. Once at the PM, Wntless is internalized by the clathrin-mediated endocytosis and is then sorted away from the lysosomal degradation route by the retromer complex [17]. This ensures retrograde transport of Wntless back to the Golgi for a subsequent round of Wnt secretion. Due to their hydrophobicity, Wnt ligands are strongly insoluble and retained bound to the PM upon secretion [18], suggesting that free Wnt diffusion is not a major player in the formation of extracellular concentration gradients.

During the last two decades, research on models organisms such as *Drosophila melanogaster* has provided an enormous advance in the understanding of how morphogen gradients are formed *in vivo*[19]. In polarized epithelial cells of *Drosophila* embryos, the fly Wnt1 homologue Wingless (Wg) is translated and secreted apically; forced basolateral targeting of Wg transcripts reduces its signaling activity [20]. Accordingly, intracellular Wg accumulates in the apical region of Wg-producing cells in the embryonic epidermis as well as epithelia of wing imaginal discs [18,21]. Therefore, it is widely accepted that Wg needs to be apically secreted from the producing cells to ensure proper signaling in the neighboring receiving cells. Yet, intriguingly, extracellular Wg is strongly concentrated at the basolateral region of producing cells, suggesting that morphogen gradients are formed basolaterally [21]. Membrane-associated Wg is actively endocytosed by the producing cells. For instance, it has been shown that ~50% of Wg-positive intracellular vesicles in the producing cells are of the endocytic nature and that they can be recycled back to the cell surface, at least to the apical PM [18]. Impairment of endocytosis in the producing cells by a dominant negative Rab5, causes excessive extracellular (both apical and basolateral) accumulation of Wg [12]. Conversely, recycling GTPases Rab4 and Rab11 appear not to be required for Wg gradient formation [12] (although all three Rabs affect Wg signal transduction in the receiving cells [22]). Interestingly, Wg is retained in the producing cells in a temperature-sensitive dynamin mutant [21]. The fact that the dynamin mutant, but not the Rab5 mutant, affects Wg secretion has been attributed to a function of dynamin, additional to its role in endocytosis—such as a role in the post-Golgi vesicle transport to the PM [23]. Cumulatively, it can be stated that although it is generally accepted that Wg is secreted apically, it remains unclear if morphogen endocytosis and recycling are required for spreading and if Wg concentration gradients are formed at the apical and/or basolateral compartments.

Heparan sulfate proteoglycans (HSPGs), which are components of the extracellular matrix, have been shown to play a role in morphogen signaling [24]. Interestingly, the glycosyl-phosphatidyl-inositol (GPI) anchored Dally-like protein (Dlp), a member of the HSPGs, has been shown to be required for the dynamin-dependent apical Wg internalization and its subsequent targeting to the basolateral compartment in the wing disc [25]. However, the Dlp-mediated Wg transcytosis from the apical to the basolateral compartment might take place mainly in the distal receiving cells, because Dlp is poorly expressed in cells within and close to the zone of Wg production [25,26]. Therefore, it has been proposed that the apical poorly diffusible pool of Wg is necessary for the activation of short-range target genes, whereas the basolateral Wg pool made by Dlp-mediated transcytosis is responsible for the expression of long-range target genes [25,27].

An alternative way to generate more diffusible Wg molecules is by their association with lipoprotein particles, which are formed by apolipoproteins inserted in a phospholipid monolayer surrounding a core of esterified cholesterol and triglycerides [28]. In *Drosophila*, lipoprotein particles are produced and secreted in the fat body, and then spread to the wing disc by the hemolymph. Knockdown of apolipophorin (the *Drosophila* homologue of vertebrate apoliproteins) reduced extracellular Wg on both the apical and basolateral epithelial regions, contracted Wg diffusion from the producing zone, and narrowed the activation of long-range but not short-range target genes [28]. Accordingly, high-density lipoproteins associate with and promote the secretion of the mammalian Wnt3a by cells in culture [29]. How Wnts can associate with lipoprotein particles is still not well-understood, however, this experimental evidence indicates that morphogens can be secreted in pools with different diffusion properties. In agreement with this view, exosomes carrying Wnt ligands produced via inward budding of multivesicular bodies (MVBs) have also been suggested as a source of morphogen diffusion over long distances. For instance, Wg-producing presynaptic cells of the *Drosophila* neuromuscular junctions (NMJs) secrete exosomal vesicles containing the morphogen into the synaptic cleft [30], a process that seems to require Rab11 [31]. Similarly, active Wnt molecules associated with exosomes are also secreted by mammalian cells and can be detected in receiving cells of the *Drosophila* wing disc [32–34]. Inhibition of exosome secretion within the Wg-producing cells of the wing disc induced intracellular Wg accumulation, reduced the extracellular pool of Wg and impaired activation of the short-range target genes [33], indicating that exosomes are not selectively responsible for the long-range Wg responses. Since PM-associated proteins contained in exosomes are mainly of the endocytic origin [35], these data suggest that apical internalization of Wg is mandatory for its inclusion in exosomes.

Recently, it has been shown that Swim, a member of the Lipocalin family of extracellular transport proteins, can interact with and facilitate the extracellular diffusion of monomeric Wg for the activation of long-range target genes in the wing disc [36]. However, Swim overexpression impaired the activation of both the short- and long-range target genes without any effect on formation of extracellular Wg gradients [36], suggesting that Swim interaction partially interferes with the ability of Wg to bind to its receptors. Likewise, secreted frizzled-related proteins (sFRPs), a family of secreted factors believed to act as Wnt antagonists [37], appear to be required for the formation of Wnt concentration gradients in the embryonic mouse optic cup [38]. In sFRP1 and sFRP2 double knockout mice, inhibition of the Wnt/β-catenin signaling was concomitant with a reduced Wnt11 spreading in the retina [38]. Additionally, forced expression of sFRP1 in the wing disc of *Drosophila*, which does not encode any sFRP homologue in its genome, expanded extracellular Wg diffusion, blocked activation of the short-range target genes, and enhanced the long-range target genes [38]. Enhanced Wnt spreading by sFRPs has been also described in *Xenopus laevis*[39], suggesting that the sFRP-mediated regulation of Wnt concentration gradients may be highly conserved among vertebrates.

We have previously identified the evolutionary conserved reggie-1/flotillin-2 (hereafter reggie-1) protein as a regulator of formation of Wg concentration gradients in the *Drosophila* wing disc [40]. We showed that the extracellular Wnt gradient is significantly narrowed upon reggie-1 knockdown, and expanded upon overexpression of reggie-1. While reggie-1 knockdown reduced the activation of the long-range target genes, its overexpression negatively affected expression of the short-range targets [40]. These effects were also observed when the reggie-1 expression levels were altered exclusively in the Wg producing cells, supporting the notion that the morphogen can be secreted in at least two different active forms: a reggie-independent poorly diffusible Wg necessary for activation of the short-target genes and a reggie-dependent highly diffusible form responsible for expression of the long-range target genes. Interestingly, the increased Wg diffusion observed upon reggie-1 overexpression was apparent exclusively at the apical region of the wing disc, indicating that Wg concentration gradients may be formed apically [40]. The molecular mechanisms involved in the reggie-mediated regulation of Wg secretion remain unstudied, but some hypotheses can be built based on the proposed functions of the reggie proteins (see below).

## 4. Reggie/Flotillin Proteins

The reggie proteins (reggie-1/flotillin-2 and reggie-2/flotillin-1) are expressed in virtually every cell type of organisms as diverse as insects and mammals [41–43]. They reside at the cytoplasmic face of the PM within local environments rich in sphingolipides and cholesterol generally known as lipid rafts [42]. They are linked to the membrane via acylations and a stretch of hydrophobic amino acids at their *N*-terminus [44], and form homo- and hetero-oligomers through their *C*-terminal regions [45]. These oligomers are thought to be the building blocks of the reggie-microdomains, a specialized type of non-caveolar lipid rafts. At the protein level, stability of the reggie proteins is interdependent, since loss of one causes reduction of the other [45,46]. Reggie-1 and -2 were initially discovered as proteins upregulated in retinal ganglion cells after optic nerve section in goldfish and rat [47,48], and found to be required for proper neuronal differentiation and axon regeneration in these animals [49–51]. Simultaneously, these proteins were discovered in the floating, detergent-resistant membrane fractions and called flotillins [52]. In addition to their role in Wnt secretion and spreading, reggies have been involved in a variety of trafficking and signaling events of membrane proteins such as epidermal growth factor receptor [53,54], cellular prion protein (PrPC) [55–57], amyloid precursor protein (APP) [58], insulin receptor [59], cholesterol transporter [60], and glutamate and dopamine transporters [61]. Moreover, reggies seem to define a specialized clathrin-independent endocytic pathway in mammalian cells [62]. Alternatively, it has been suggested that reggie-microdomains may be required for the targeted delivery of particular membrane proteins from intracellular compartments to specific sites of the PM [63].

How reggies affect Wnt secretion and spreading is not yet understood, but available data provide some interesting indications. For instance, reggie-microdomains (lipid rafts) seem to follow the same intracellular routes as Wnts: from the endoplasmatic reticulum to Golgi and then to the PM [64]. Since Wnts associate with lipid rafts [65] and the latter are known to assist vesicle transport to the PM [66], it is tempting to speculate that the reggie-microdomains may play a direct role in Wnt trafficking and secretion. Accordingly, overexpression of reggie-1 increased Wg secretion in *Drosophila* S2 cells, while expression of the truncated dominant negative form of reggie-1 had the opposite effect [40]. Since Wg secreted from the reggie-1-overexpressing cells became more uptakable by surrounding S2 cells and more diffusible in the wing disc [40], reggies may determine a specialized secretion route. This specialized route may require endocytosis and recycling of Wg molecules in the producing cells. Reggie is known to control clathrin- and caveolin-independent endocytosis of GPI-anchored proteins in HeLa cells [62] and in basolateral membranes of polarized hepatic cells [67]; in the latter system, reggie was shown to cooperate with dynamin [67]. In some polarized epithelial cells (e.g., hepatocytes), various GPI-anchored proteins are first secreted basolaterally followed by endocytosis and specific sorting towards the apical compartment [67,68]. As mentioned above, extracellular Wg is accumulated at the basolateral site of the producing cells, and a dynamin mutant negatively affects Wg secretion. Therefore, it is plausible that a dynamin- and reggie-dependent endocytosis of basolateral Wg followed by its apical re-secretion may generate a specialized diffusible pool of the morphogen. To date, it is unknown if the reggie-mediated endocytosis is dependent on Rab5. In case it is not, this might explain the different effects of the dynamin and Rab5 mutations on Wg secretion [12,21].

**Figure 1:**
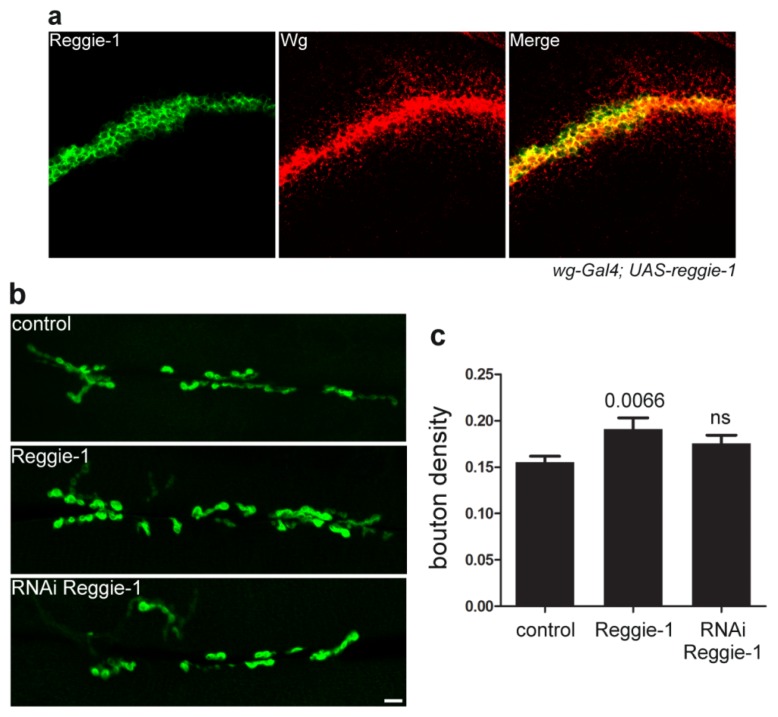
Reggie-1 is not secreted together with Wg in the *Drosophila* wing disc (**a**) Overexpression of reggie-1 was induced in the Wg producing cells using the driver *wg-Gal4.* Immunostaining against Wg shows formation of the Wg gradient in the wing disc, with the morphogen diffusing away from the zone of production. In contrast, reggie-1 is absent in the Wg-receiving cells, indicating that it is not significantly secreted by the producing cells. The *Drosophila* stocks and antibodies were used as described [40]; (**b**) Reggie-1 does not play a significant role in NMJ formation in *Drosophila*. Reggie-1 overexpression or downregulation by RNAi (obtained from the Vienna Drosophila RNAi center) in motoneurons was achieved using the driver *OK371-Gal4*[71]. The postsynaptic membrane of the NMJs is visualized using the construct CD8-GFP-Sh which accumulates in the subsynaptic reticulum [72]. Representative confocal images of the NMJs overexpressing or downregulating reggie-1 show no obvious morphological phenotypes compared to controls. Scale bar: 10 μm; (**c**) Quantification reveals that overexpression of reggie-1 results in a slight increase in bouton (synapse) density compared to controls (*p*-value by the Student’s *t*-test), while no significant (ns) effect can be observed for the reggie-1-depleted NMJs.

The role of reggie-1 in morphogen secretion and spreading is not restricted to Wg—it additionally controls spreading of and signaling by Hedgehog [40]. On the other hand, reggie-1 appears to be specific for lipid-modified morphogens, as it does not affect spreading of the lipid-unmodified Dpp [40]. Interestingly, Hedgehog signaling and spreading is also affected by its association with lipoprotein particles [28,69] and Dlp [25], but neither with exosomes [33] nor Swim [36]. Since Dlp is poorly expressed in the Wg-producing cells, while effects of reggie-1 require its expression in these cells, it is rather unlikely that reggie-1 may be involved in Dlp functions as suggested previously [6,27]. Although reggies localize in MVBs and are commonly used as markers of exosomes [35], a role of reggie-1 in exosomal Wg secretion and spreading is probably minor. For instance, reduced Wg secretion in exosomes does not specifically affect long-range target genes in the wing disc [33], and we show that reggie-1 is undetectable in the Wg-receiving cells after its overexpression in the producing cells, in contrast to Wg itself (Figure 1a). Moreover, our analysis shows that reggie-1 down- or over-expression in motoneurons does not have a strong impact on NMJ formation in *Drosophila* (Figure 1b,c), conversely to the effects seen upon Wg overexpression [70] or to the activation of the Wg-mediated downstream signaling (V.L.K. and A-M.L. unpublished data). Since Wg is secreted in exosomal vesicles at the NMJs [30], these observations are not in agreement with a function of the reggie proteins in the Wg secretion in exosomes.

The striking similarity between the Wg phenotypes observed upon genetic manipulations of apolipoprotein and reggie-1 suggests that these two molecules are functionally related. Notably, lipoprotein receptors and the Hedgehog receptor Patched (Ptc) are required to package Hedgehog onto lipoprotein particles which are, in turn, responsible for spreading of the morphogen [69]. Association of Hedgehog with the lipoprotein particles seems not to require endocytosis [69], suggesting that extracellular PM-bound Hedgehog is sequestered by the particles through lipoprotein receptor-mediated recruitment. Likewise, it has been suggested that the lipoprotein particle-dependent spreading of Wg involves its co-receptor Arrow (LRP5/6 in mammals) [69], which is a member of the low-density lipoprotein receptor-related protein (LRP) family. Therefore, we alternatively envision that the reggie-microdomains at the apical PM may create a “dating point” for morphogens and lipoprotein receptors which specifically allows morphogen sequestration via lipoprotein particles. In agreement with this view, members of the LRP family have been shown to transiently associate with lipid rafts [73,74] and to be involved in the trafficking of the raft-associated proteins PrPC [75] and APP [76]—proteins known to reside in reggie-microdomains [57,58].

**Figure 2:**
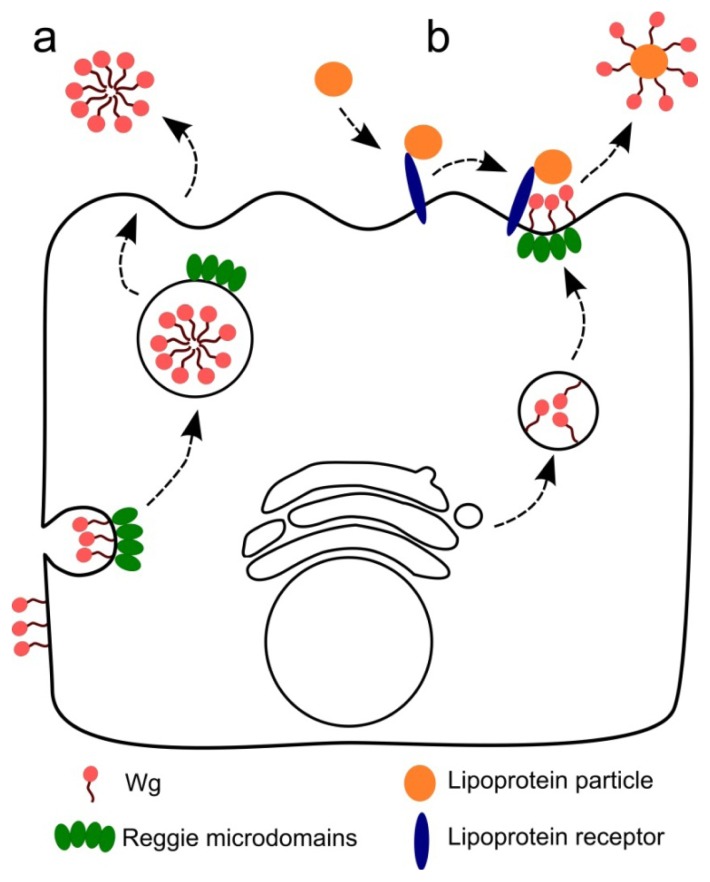
Model for the possible roles of reggie/flotillin proteins in formation of the highly diffusive pool of Wingless. (**a**) Wingless (Wg), accumulating at the basolateral site of the Wg-producing cells, can be endocytosed by a reggie-dependent mechanism and re-secreted apically. During the processes of reggie-dependent endocytosis and intracellular trafficking, Wg is packaged into a highly diffusive form; (**b**) Reggie-microdomains serve as the “dating points” where apically secreted Wg “meets” with the lipoprotein receptors and particles, permitting loading of the morphogens onto the lipoprotein particles for high-range diffusion.

Altogether, we propose two models to explain the role of the reggie proteins in Wg spreading and long-range signaling (Figure 2). The first model includes a dynamin- and reggie-dependent endocytosis of extracellular Wg from basolateral membranes followed by sorting and secretion to the apical compartment of the producing cells (Figure 2a). During apical sorting, Wg molecules may be “prepared” to ensure long-range diffusion for the activation of long-range target genes. This scenario may involve formation of micelle-like aggregates that shield the hydrophobic lipid adducts in their interior as previously reported for Hedgehog [77]. The second model (Figure 2b) describes the apical reggie-microdomains as “dating points” in the producing cells where extracellular Wg transiently interacts with lipoprotein receptors (such as Arrow) to allow Wg sequestration via lipoprotein particles. Then, lipoprotein particles containing Wg can diffuse over long distances within the apical milieu of the wing disc to guarantee long-range responses. Both models presume that reggies are required in the producing cells to generate a highly diffusible pool of morphogens and that concentration gradients are formed at the extracellular apical region of the wing disc.

## 5. Wnt, Reggies and Breast Cancer

The fact that Wnt morphogens are involved in cancer was discovered 30 years ago, when the first Wnt gene was identified as the integration site of the mouse mammary tumor virus [78]. To date, overexpression of various canonical Wnt ligands as well as mutations activating downstream components of the canonical pathway have been uncovered in a wide range of cancers and various human diseases [2–4]. Excessive activation of the β-catenin-mediated transcription seems to be one of the major players in the early stages of cancer development [2–4]. Accordingly, approximately 50% of breast cancers are linked to overactivation of the canonical Wnt pathway [79] and a wide range of breast cancer cell lines display high expression levels of canonical Wnt ligands and receptors [80], including the most aggressive and therapeutically intractable triple-negative breast cancer [81]. On the other hand, the role of the non-canonical Wnt pathway in cancer has not been well characterized, but an increasing amount of studies pointed to this pathway as a key element during later stages of cancer progression, such as invasiveness and metastasis [82]. For instance, overproduction of non-canonical Wnt ligands has been observed in brain metastases derived from breast tumors [83]. Thus, understanding how Wnt ligands are secreted and how extracellular gradients are formed will provide the much needed knowledge required to identify new drug targets to combat breast cancer.

**Figure 3:**
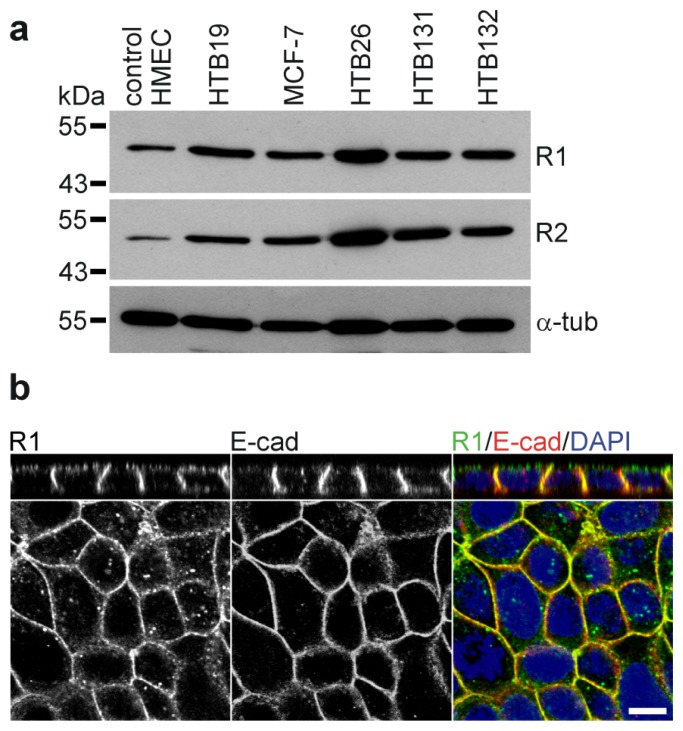
Reggie expression in breast cancer cell lines. (**a**) Western blot analysis from total cell extracts were done as previously reported [45] revealing a stronger expression of reggie-1 (R1) and reggie-2 (R2) in the breast cancer cell lines HTB19, MCF-7, HTB26, HTB131 and HTB132 as compared to normal human mammary epithelial cells (HMEC). Tubulin was probed as a loading control (α-tub); (**b**) Polarized MCF-7 cells were immunostained against reggie-1 and E-cadherin (E-cad) as described previously [53]. Confocal images showed localization of reggie-1 at intracellular compartments as well as at the apical and lateral membranes. The cell adhesion molecule E-cadherin was used as control and is mainly localized at lateral membranes. XZ sections are shown in the upper panels. Scale bar: 10 μm.

Only recently, a potential role of the reggie proteins in cancer has emerged. For instance, upregulation of reggie proteins has been observed in tumorigenic and metastatic melanoma cell lines [84] and in esophageal squamous cell carcinomas [85]. Moreover, high levels of reggie expression were detected in various breast cancer specimens and derived cell lines [86–88] (Figure 3a). Breast cancer tissues show a significant correlation between poor patient survival time and the expression levels of reggie-1 and reggie-2 [87,88]. Notably, downregulation of reggie-1 or -2 reduced proliferation and the tumorigenicity and metastatic capability of human breast cancer cell lines *in vitro* and *in vivo*[86,87]. The first attempts to elucidate the molecular mechanisms involved in these processes implicate the Akt cell survival signaling pathway [87,88]. For example, knockdown of reggie-2 in the MCF-7 and HTB26 breast cancer cell lines affects proliferation and tumorigenicity by a suppression of the Akt signaling pathway which, in turn, enhances the activity of the transcription factor FOXO3a [87]. Similarly, reggie-1 depletion reduced the activation levels of Akt in the HTB30 breast cancer cell line [88]. Interestingly, a reggie-mediated PM-stabilization of the tyrosine kinase receptor ErbB2, which is a predictor of poor prognosis in breast cancer patients, seems to be the upstream regulator of the Akt signaling in this cell line [88]. Therefore, breast cancer cells continuously activate the Akt cascade due, in part, to impaired internalization and degradation of the activated ErbB2 receptor caused by the high reggie protein levels.

Although the molecular mechanisms behind the role of reggies in breast cancer development are not fully understood, their connection to Wnt secretion, spreading and signaling appears as a promising new avenue for further studies. For instance, polarized epithelial cell lines may represent a simple and versatile *in vitro* model to analyze Wnt secretion in a more physiological way than the non-polarized cell systems commonly used for such studies. Some breast cancer cell lines such as the MCF-7 are able to form a polarized cell monolayer on filters [89], representing a suitable model not only for the analysis of Wnt polarized secretion and signaling but also to elucidate the role of the reggie proteins in these processes. Interestingly, endogenous reggie-1 localization in polarized MCF-7 cells is restricted to intracellular vesicles as well as to the lateral and apical membranes (Figure 3b), which is in agreement with a potential role of reggies in polarized membrane protein trafficking. Understanding the exact role of reggies in Wnt secretion, spreading and signaling as well as in breast cancer progression, will not only increase our knowledge of the biology behind these important phenomena, but may also allow the development of novel therapeutic strategies.
